# Supplementary material for: Evaluation of Latent Models Assessing Physical Fitness and the Healthy Eating Index in Community Studies: Time-, Sex-, and Diabetes-Status Invariance
Source: Nutrients. 2021 Nov 26;13(12):4258. doi: 10.3390/nu13124258 (PMC8708138; doi:10.3390/nu13124258)
Supplement: Supplementary file 1 [file nutrients-13-04258-s001.zip › nutrients-1427409-supplementary.pdf]

Supplement to Measurement Paper

Table S1. Age and sex specific criteria to achieve maximum scores for each HEI-C component

	Criteria for maximum score			
	**Male 19-50 yo**	**Male 51+ yo**	**Female 19-50 yo**	**Female 50+ yo**
**Adequacy**				
Total vegetables and fruit[1] Maximum score = 10	8	7	7	7
Whole fruit[1] Maximum score = 5	2	1.5	1.5	1.5
Dark green and orange vegetables[1] Maximum score = 5	2	1.5	1.5	1.5
Total grain products[1] Maximum score = 5	8	7	6	6
Whole grains[1] Maximum score = 5	4	3.5	3	3
Milk and alternatives[1] Maximum score = 10	2	3	2	3
Meat and alternatives[1] Maximum score = 10	3	3	2	2
Unsaturated Fats[1] Maximum score = 10	3	3	2	2
**Moderation**				
Saturated fats[2] Maximum score = 10	7%	7%	7%	7%
Sodium[3] Maximum score = 10	1500 mg	1500 mg	1500 mg	1500 mg
"Other food" [2] Maximum score = 20	5%	5%	5%	5%

[1]Number of servings recommended in *Eating Well With Canada's Food Guide* [50]
[2]Percent of total energy
[3]Scores for sodium based on Dietary Reference Intakes

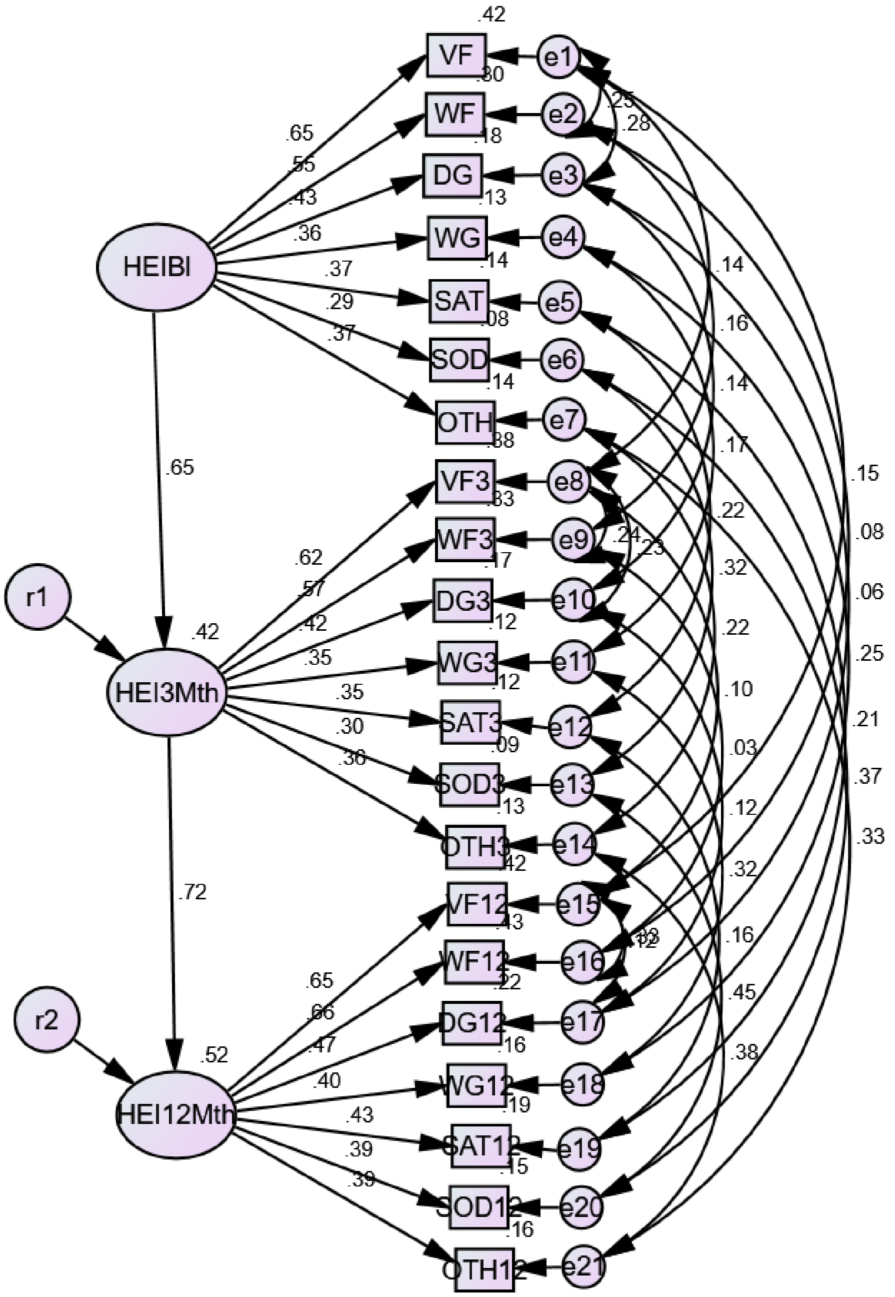

Figure S1. Longitudinal Invariance of Reduced HEI-C Model. HEIBl=total HEI-C baseline; HEI3Mth= total HEI-C at 3-months; HEI12Mth=total HEI-C at 12-months; VF=total vegetables and fruit; WF=whole fruit; DG= dark green and orange vegetables; WG= whole grains; SF=saturated fats; SOD=sodium; OTH= Other foods; e# and r#=error terms. Squares are measured variables; circles are latent variables.

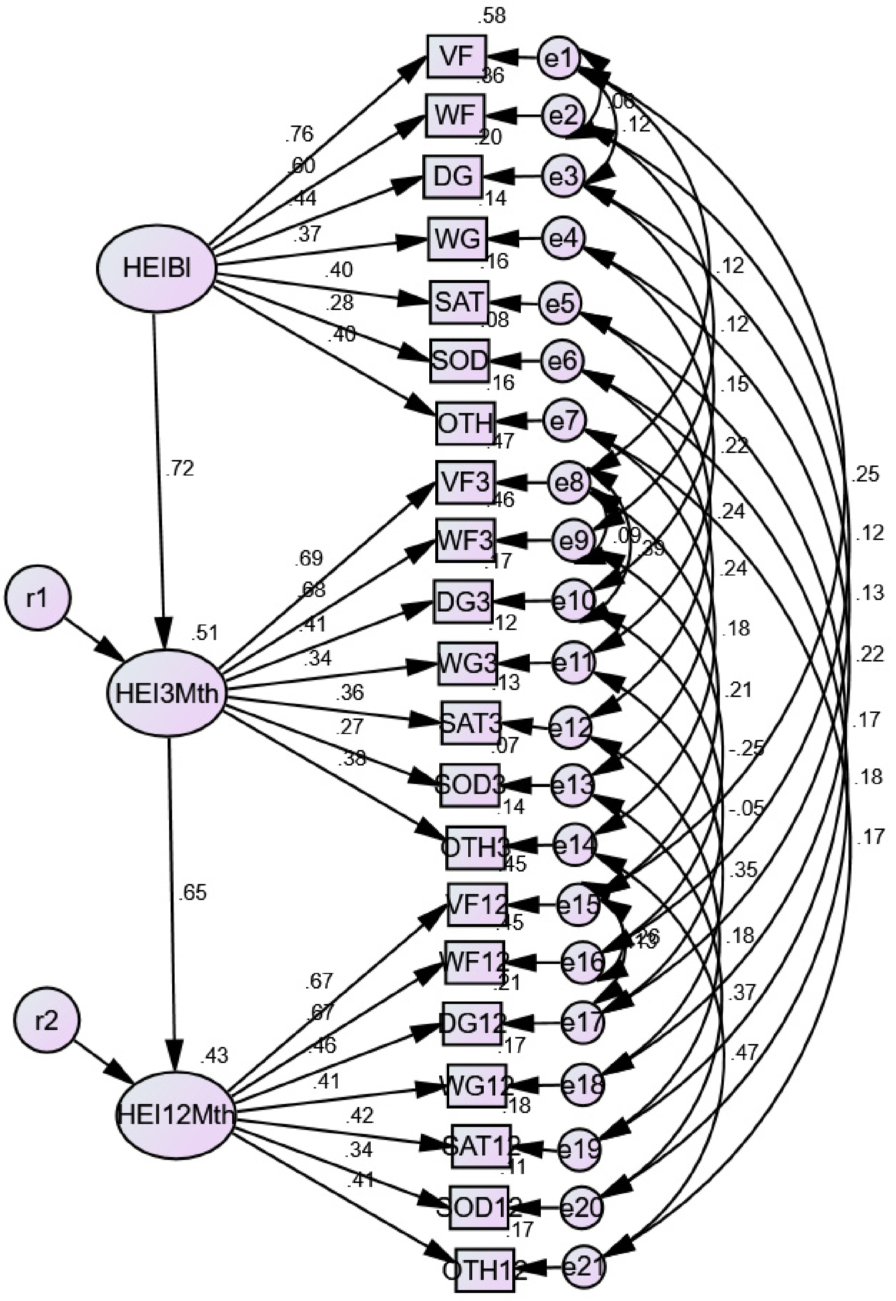

Figure S2. Sex Invariance of Reduced HEI-C Model. HEIBl=total HEI-C baseline; HEI3Mth= total HEI-C at 3-months; HEI12Mth=total HEI-C at 12-months; VF=total vegetables and fruit; WF=whole fruit; DG= dark green and orange vegetables; WG= whole grains; SF=saturated fats; SOD=sodium; OTH= Other foods; e# and r#=error terms. Squares are measured variables; circles are latent variables.

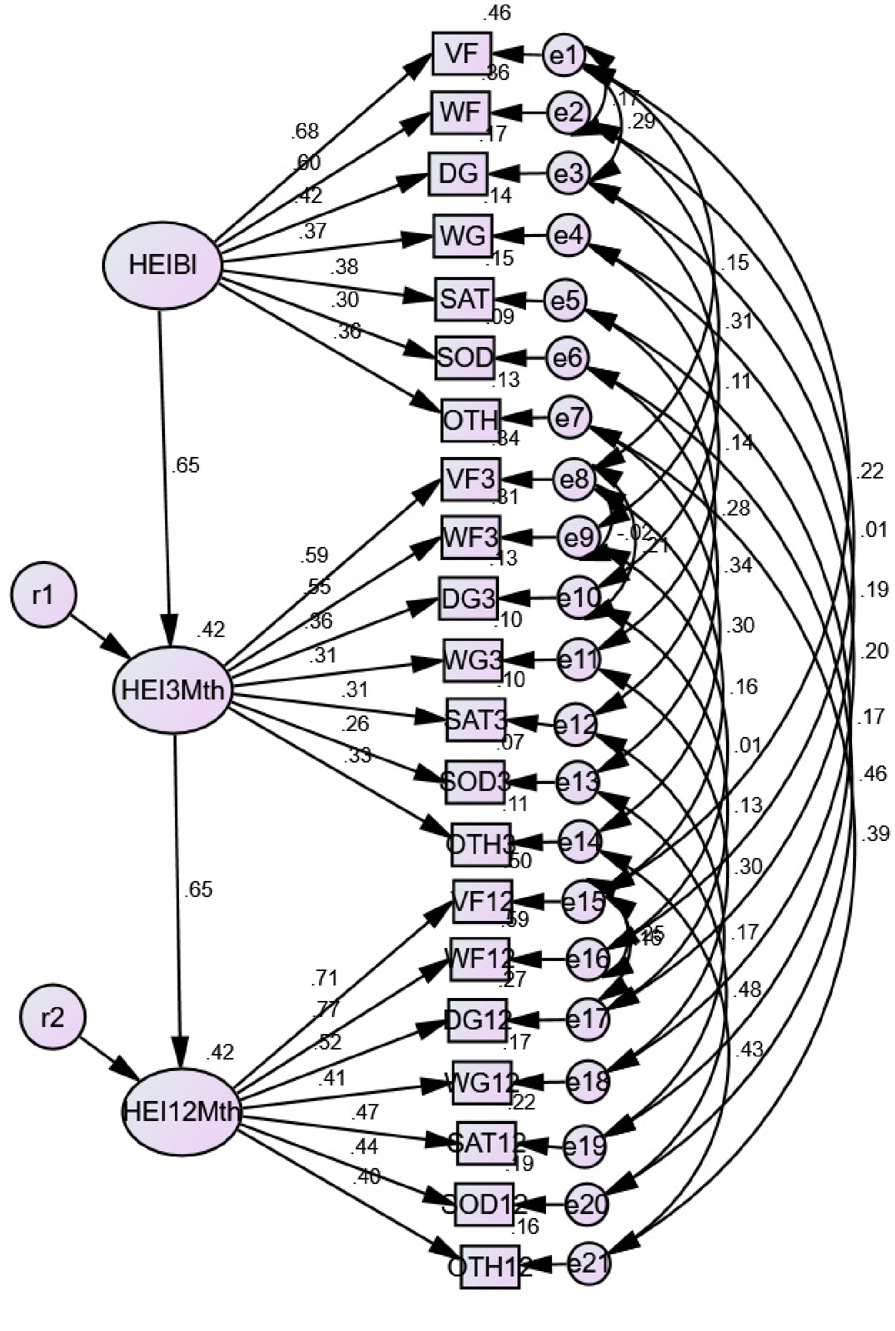

Figure S3. Disease-State Invariance of Reduced HEI-C Model. Loadings are identical across the three time points and across disease-states (noDM and DM). HEIBl=total HEI-C baseline; HEI3Mth= total HEI-C at 3-months; HEI12Mth=total HEI-C at 12-months; VF=total vegetables and fruit; WF=whole fruit; DG= dark green and orange vegetables; WG= whole grains; SF=saturated fats; SOD=sodium; OTH= Other foods; e# and r#=error terms. Squares are measured variables; circles are latent variables.

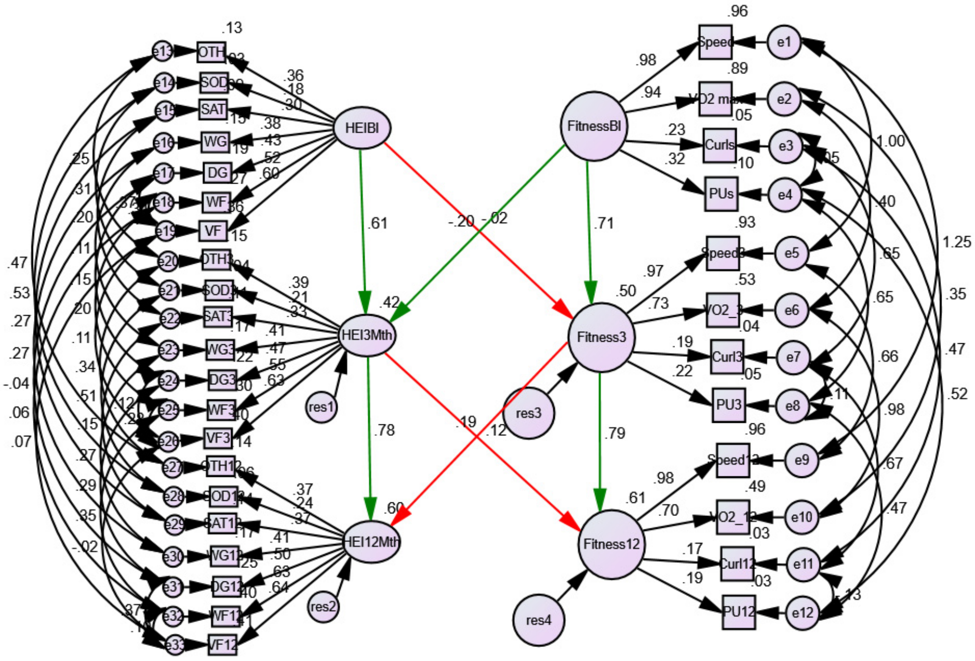

Figure S4. Results for women showing structural regressions between HEI and Fitness factors. Regression paths in green show significant results, structural regression in red were not statistically significant. HEIBl=total HEI-C baseline; HEI3Mth= total HEI-C at 3-months; HEI12Mth=total HEI-C at 12-months; VF=total vegetables and fruit; WF=whole fruit; DG= dark green and orange vegetables; WG= whole grains; SF=saturated fats; SOD=sodium; OTH= Other foods; e# and r#=error terms. Squares are measured variables; circles are latent variables.

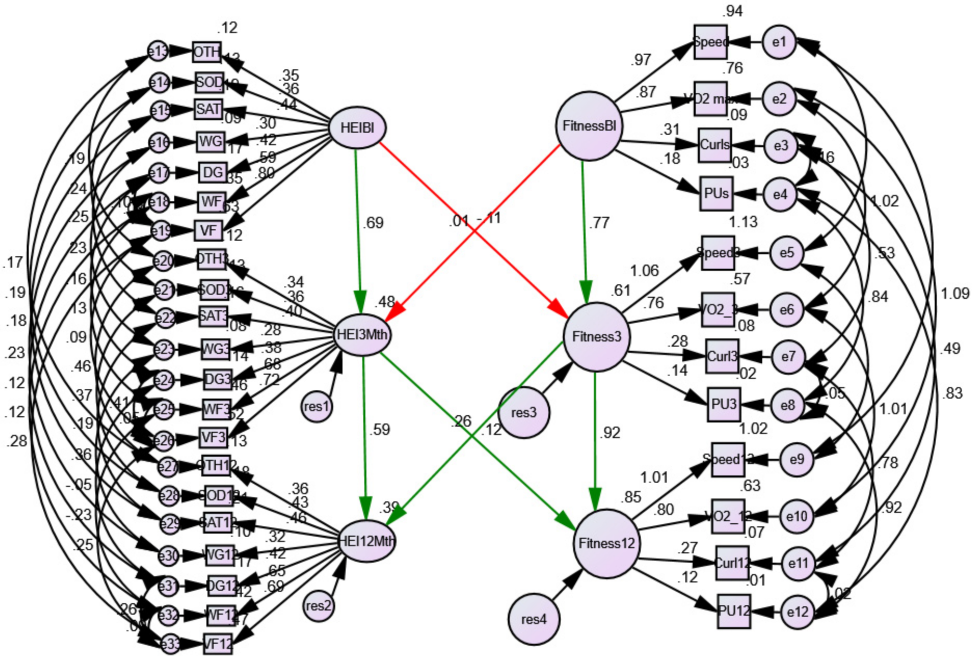

Figure S5. Results for men showing structural regressions between HEI and Fitness factors. Regression paths in green show significant results, structural regression in red were not statistically significant. HEIBl=total HEI-C baseline; HEI3Mth= total HEI-C at 3-months; HEI12Mth=total HEI-C at 12-months; VF=total vegetables and fruit; WF=whole fruit; DG= dark green and orange vegetables; WG= whole grains; SF=saturated fats; SOD=sodium; OTH= Other foods; e# and r#=error terms. Squares are measured variables; circles are latent variables.

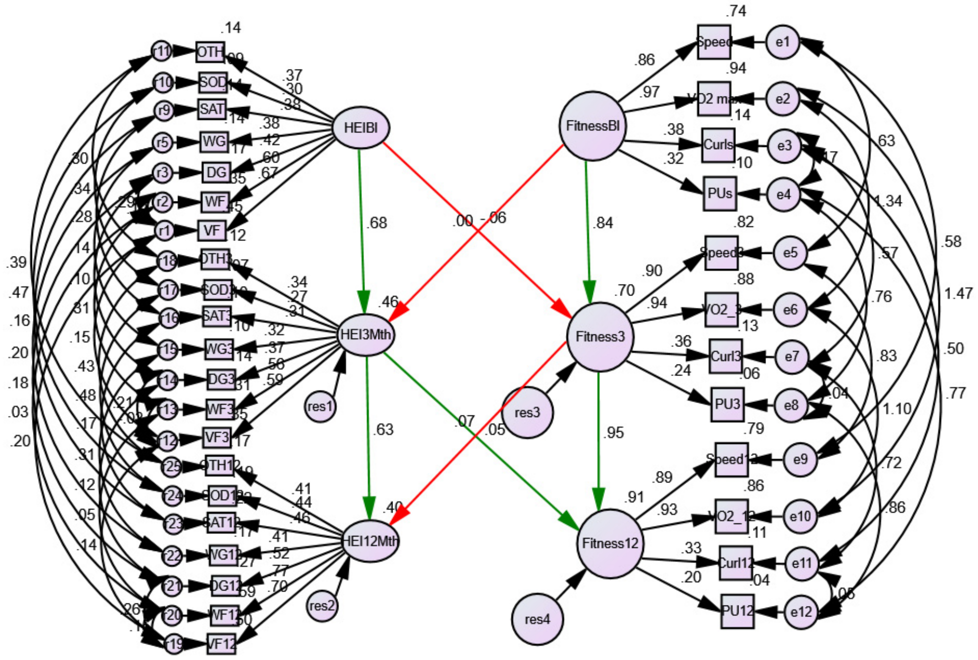

Figure S6. Overall disease and time invariant results showing structural regressions between HEI and Fitness factors. Regression paths in green show significant results, structural regression in red were not statistically significant. HEIBl=total HEI-C baseline; HEI3Mth= total HEI-C at 3-months; HEI12Mth=total HEI-C at 12-months; VF=total vegetables and fruit; WF=whole fruit; DG= dark green and orange vegetables; WG= whole grains; SF=saturated fats; SOD=sodium; OTH= Other foods; e# and r#=error terms.
